# Supplementary material for: Modelling Skylarks (Alauda arvensis) to Predict Impacts of Changes in Land Management and Policy: Development and Testing of an Agent-Based Model
Source: PLoS One. 2013 Jun 6;8(6):e65803. doi: 10.1371/journal.pone.0065803 (PMC3675089; doi:10.1371/journal.pone.0065803)
Supplement: Supporting Information S4 — The skylark ODdox as a zipped archive. (ZIP) [file pone.0065803.s004.zip › Skylark_ODdox/class_roe_deer_info.html]

ALMaSS Skylark ODdox: RoeDeerInfo Class Reference


ALMaSS Skylark ODdox
 2.0


- Main Page
- Related Pages
- Classes
- Files

- Class List
- Class Index
- Class Hierarchy
- Class Members

Public Attributes

RoeDeerInfo Class Reference

Part of the basic ALMaSS system (obselete)
More...

`#include <populationmanager.h>`

List of all members.

|  |  |
| --- | --- |
| Public Attributes | |
| int | m\_Age |
| unsigned | m\_OldRange\_x |
| unsigned | m\_OldRange\_y |
| unsigned | m\_Range\_x |
| unsigned | m\_Range\_y |
| int | m\_Size |
| Public Attributes inherited from AnimalPosition | |
| TTypesOfLandscapeElement | m\_EleType |
| TTypesOfVegetation | m\_VegType |
| unsigned | m\_x |
| unsigned | m\_y |

---

## Detailed Description

Part of the basic ALMaSS system (obselete)

Communicates the range centre, age and size of animals to other objects

---

## Member Data Documentation

int RoeDeerInfo::m\_Age

unsigned RoeDeerInfo::m\_OldRange\_x

unsigned RoeDeerInfo::m\_OldRange\_y

unsigned RoeDeerInfo::m\_Range\_x

unsigned RoeDeerInfo::m\_Range\_y

int RoeDeerInfo::m\_Size

---

The documentation for this class was generated from the following file:

- populationmanager.h


- RoeDeerInfo
- Generated on Thu Jan 10 2013 13:15:37 for ALMaSS Skylark ODdox by
   1.8.1.1
